# Clinical predictors of residual disease in hysterectomy following a loop electrosurgical excision procedure for cervical intraepithelial neoplasia grade 3

**DOI:** 10.1186/s12884-022-05281-y

**Published:** 2022-12-27

**Authors:** Qing Wu, Yu Jiang, Jun Ding, Lihua Xia, Haiou Xu

**Affiliations:** 1 Center of Reproductive medicine, Department of Gynecology, Affiliated People’s Hospital, Zhejiang Provincial People’s Hospital, Hangzhou Medical College, 310014 Hangzhou, Zhejiang, P.R. China; 2 Department of Obstetrics and Gynecology, Fujian Provincial Hospital, Clinical Medical School of Fujian Medical University, 350001 Fuzhou, Fujian P.R. China; 3 Cervical Disease Center, Department of Obstetrics and Gynecology, Hangzhou Women’s Hospital (Hangzhou Maternity and Child Health Care Hospital), 310014 Hangzhou, Zhejiang, P.R. China

**Keywords:** Conization, Cervical intraepithelial neoplasia, Transformation zone, Human papillomavirus

## Abstract

**Objective:**

To investigate the predictors of residual disease in a hysterectomy following a loop electrosurgical excision procedure (LEEP) for cervical intraepithelial neoplasia (CIN) 3.

**Methods:**

This retrospective study identified 421 patients with histologically confirmed CIN 3 who underwent LEEP and subsequently had a hysterectomy within 6 months. The clinical data included age, parity, type of transformation zone, cytology results, human papillomavirus (HPV) genotype test, endocervical curettage (ECC), and pathological data of LEEP and hysterectomy were obtained from the medical records. A logistic regression model was used to analyze the relationship between the variables and the risk of residual disease in the hysterectomy samples.

**Results:**

186 (44.18%) patients had residual disease in the hysterectomy specimens. The predictive markers of residual disease following LEEP included positive ECC, positive margin of the samples from LEEP, type II or III transformation zone, HPV16 and HPV18 infection, and other high-risk HPV. HPV-18 positivity (OR, 7.13; 95% CI, 3.49 to 14.56; *p* < 0.001) and type III transformation zone (OR, 6.37; 95% CI, 2.91 to 13.94; *p* < 0.001) were the most indicative of residual disease following LEEP.

**Conclusion:**

Positive high-risk HPV, particularly HPV18, positive ECC, the positive margin of specimens from LEEP, and type II or III transformation zone were reliable prognostic markers of residual disease following a LEEP for CIN 3.

## Introduction

Most CIN 1 lesions regress spontaneously and may less frequently progress to CIN 2–3 [1, 2]. CIN 2–3 are considered high-grade lesions and have a more significant potential for evolving into an invasive cervical carcinoma [3]. Cervical intraepithelial neoplasia 3 (CIN3) is a premalignant state of cervical cancer and is mainly associated with human papillomavirus (HPV) infection.

Given the high risk of progression to cervical cancer of CIN 3, prompt treatment is recommended. Conization is the recommended treatment for CIN 2–3 usually and rules out microinvasive cancer or worse not detected before. It can be performed by cold knife conization, laser conization, loop LEEP or large loop excision of the transformation zone [4, 5]. Most clinicians prefer the LEEP as this office procedure is easily performed and allows deep excision of the cervical transformation zone with minimal damage. This simple procedure also has a shorter operative time and low cost [5–7].

However, conization may result in incomplete eradication with significantly higher risks of residual or recurrent lesions, especially for CIN 3 [8]. Studies have reported residual disease or recurrence rates of CIN 2 + after conization ranging from 1.1–17.7% [8, 9]. Residual intraepithelial neoplasia after incomplete excision by conization of CIN 3 was found in 29–57% of patients who subsequently underwent hysterectomy [9, 10].

Hysterectomy is not currently recommended to all women with involved margins after LEEP of CIN 3. Predicting residual uterine lesions can help doctors and patients decide the next treatment. This study aimed to determine the characteristics associated with residual lesions in the hysterectomy specimens and predict the presence of residual disease following LEEP for CIN 3.

## Materials and methods

This retrospective study was approved by the ethics committee of Zhejiang Provincial People’s Hospital (NO.2021QT047). This study included patients with histologically confirmed CIN 3 who underwent LEEP and further underwent a hysterectomy within 6 months, from January 2012 to December 2021, in the Department of Gynecology of Zhejiang Provincial People's Hospital and Department of Obstetrics and Gynecology of Fujian Provincial Hospital. Clinical data such as age, parity, type of transformation zone, cytology results, HPV genotype test, endocervical curettage (ECC), and pathological data of LEEP and hysterectomy were obtained from the medical records. Women with fertility requirement, desire of uterine sparing or incomplete clinical documents were excluded from this study (*n* = 636).

The patients underwent a laparoscopic hysterectomy after LEEP for CIN 3 based on evaluation of the difficulty in secondary lesion excision, whether to receive strict follow-up, the uterus and fertility sparing requirements, patients’ attitudes and other indications for hysterectomy such as uterine myoma, adenomyosis et al. The hysterectomy was indicated in 138 patients due to positive cone margins with high difficulty of secondary excision and in 127 patients for other gynecological diseases such as uterine myoma or adenomyosis. Hysterectomy was also proposed in 156 patients with poor follow-up and patients with emotional distress over the possibility of occult invasive cervical cancer. In this study, residual disease was defined as the presence of CIN 2, CIN 3, cervical carcinoma in situ, or invasive cancer in the hysterectomy specimens following LEEP.

### Surgical procedures

LEEP was performed with a loop electrode and electrosurgical unit in a blended model consisting of a 50W cutting current and 30 W coagulation, and we tried to remove the lesion as a whole. In the event of a bleed, electrocoagulation was performed until hemostasis was achieved. In preparation for LEEP, Lugol's iodine solution was applied under colposcopy to evaluate the extent of the cervical lesions and estimate the circumference and width of the excision. After visualization of the cervix and the squamocolumnar junction (SCJ), the transformation zone (TZ) was assessed in its native condition (type I: TZ fully visible; type II: TZ completely visible inside the endocervical canal; type III: TZ not visible). The length of excision was determined based on the transformation zone. A 3–5mm ectocervical resection margin was typically respected. If the TZ was fully observed, a length of 1cm was removed from the cervix. At least, 1.5cm and 2cm were removed for TZ types II and III, respectively. Adequate cone width was defined as a margin of 3-5mm outside the transformation zone.

After cervical conization, the specimen was marked at the 12 o'clock position, and each sample was measured to determine the length and width before fixation. Two pathologists confirmed all the histopathology reports of the whole LEEP specimens. The margins of the specimens included: the endocervical margin (inner side of the incision and the deep margin) and the ectocervical margin. Patients with histologically confirmed CIN 3 underwent a hysterectomy within 6 months after LEEP. The surgical procedure consisted of a standardized total laparoscopic hysterectomy.

### Statistical analysis

The results were reported as mean ± standard deviation for continuous variables; categorical data were presented as the count and percentage. Before the two-sample t-test, the examined features had been confirmed to conform to a normal distribution. Continuous variables such as age were analyzed with two-sample t-tests. The Chi-square test was used to compare categorical data such as the status of margin and ECC. A logistic regression model was used to analyze the relationship between the variables and the probability of residual disease in subsequent hysterectomy samples and build the clinical prediction model. A receiver operating characteristic (ROC) curve was used to determine the most appropriate cut-off value of the factors to predict residual disease. SPSS 17.0 software (SPSS Inc., Chicago, IL, USA) was used for statistical analysis. All the reported p-values were two-sided, and *p*-values < 0.05 were considered statistically significant.

## Results

Residual disease was found in 186 (44.18%) of the hysterectomy specimens. The residual disease was classified as CIN 2 in 21 patients, CIN 3 in 150 patients, adenocarcinoma in situ in 3 patients, and IA1 cancer (including endocervical adenocarcinoma) in 12 patients. The overall HPV-positive rate was 96.67% (407/421).

The clinical characteristics of the 421 eligible patients are listed in Table 1. The age of the patients, gravidity and parity of the two groups were not significantly different. However, significant differences were observed in the ECC positive rate, the distribution of the transformation zone, and the positive margin rate of the specimens from LEEP between the two groups (*p* < 0.05). The ECC positive rate and positive margin rate of the residual disease group were higher than the rates of the group without residual disease (29.03% vs. 14.04%; 44.62% vs. 23.40%; *p* < 0.05), and the transformation zone in the residual lesion group was more likely type III. The HPV genotype test showed that the positive rate of HPV16 between the two groups was not significantly different, while the positive rate of HPV 18 and other high-risk HPV were considerably higher in the residual disease group (24.73% vs. 8.94%; 39.78% vs. 23.83%; *p* < 0.001).

**Table 1:** Clinical and pathological characteristics

		Present residual disease (*n* = 186)	Absent residual disease(*n* = 235)	T/χ2	*P*-value
Age (yr)		52.68 ± 8.63	58.351 ± 8.35	1.54	0.12
Gravidity (n)		2.64 ± 0.93	2.66 ± 0.84	0.23	0.82
Parity (n)		1.27 ± 0.48	1.34 ± 0.53	1.41	0.16
HPV genotype	HPV16	98	112	1.05	0.30
	HPV18	46	21	19.36	0.00
	Other high-risk HPV	74	56	12.38	0.00
Cytology results	NILM	6	5	5.16	0.27
	ASCUS	18	39		
	LSIL	21	25		
	ASC-H	17	25		
	HSIL	124	141		
Colposcopy	LSIL	9	8	0.55	0.46
	HSIL	177	227		
ECC	Negative	132	202	14.23	0.00
	Positive	54	33		
Transformation zone	I	11	45	29.75	0.00
	II	20	51		
	III	155	139		
Margins of the specimens from LEEP	Positive	83	55	21.22	0.00
	Negative	103	188		

*HPV* Human papillomavirus, *ECC* Endocervical curettage, *LEEP *Loop electrosurgical excision procedure

The odds ratio (OR) was adjusted for covariates in the multivariate logistic regression model. According to this analysis, the reliable predictive markers of residual disease following LEEP included positive ECC, positive margin of the specimens from LEEP, type II or III transformation zone, positive HPV16, HPV18, and other high-risk HPV (Table 2). HPV-18 positivity (OR, 7.13; 95% CI, 3.49 to 14.56; *p* < 0.001) and type III transformation zone (OR, 6.37; 95% CI, 2.91 to 13.94; *p* < 0.001), as displayed in Table 2. According to the clinical prediction model, each variable was given a risk score (HPV16 positivity, 6; HPV-18 positivity, 20; other high-risk HPV positivity, 9; positive ECC, 6; type II Transformation Zone, 6; type III transformation zone, 18; positive margins of the specimens from LEEP, 10) to predict the risk of residual disease following LEEP for CIN 3.

**Table 2:** A logistic regression model was used to analyze the relationship between variables and the probability of residual disease in subsequent hysterectomy samples

		B	SE	Wald X^2^	*P*-value	OR	95%CI
HPV genotype	HPV16	0.58	0.24	5.63	0.02	1.79	1.11–2.88
	HPV18	1.96	0.36	29.10	0.00	7.13	3.49–14.56
	Other high-risk HPV	0.88	0.26	11.78	0.00	2.41	1.46–3.99
ECC	Positive	0.64	0.28	5.15	.023	1.90	1.09–3.3
Transformation zone	II	0.58	0.47	1.55	0.21	1.79	0.72–4.48
	III	1.85	0.40	21.48	0.00	6.37	2.91–13.94
Margins of the specimens from LEEP	Positive	1.02	0.24	17.62	0.00	2.77	1.72–4.46

*HPV* Human papillomavirus, *ECC* Endocervical curettage, *LEEP* Loop electrosurgical excision procedure

**Fig. 1:**
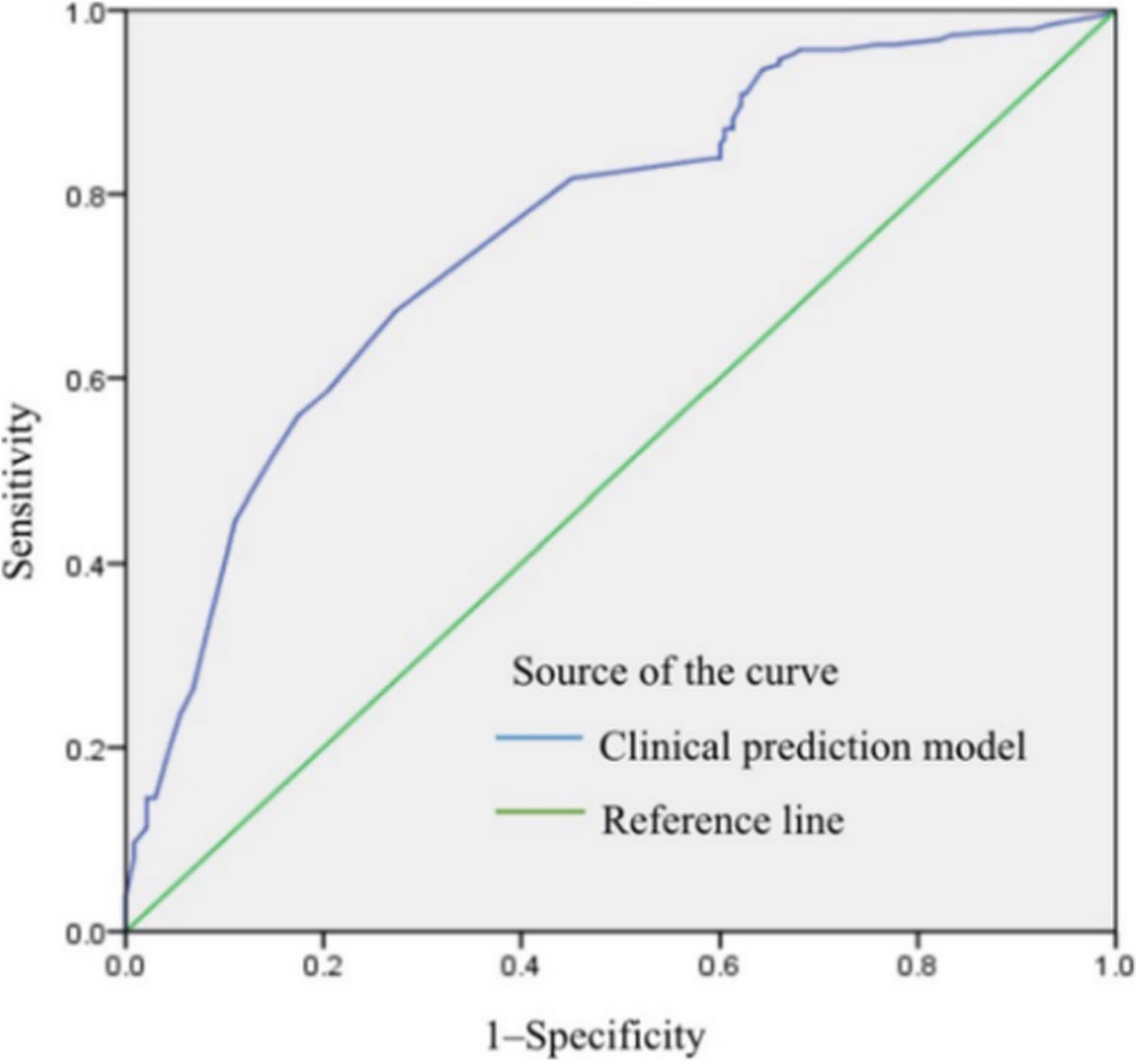
Receiver operating characteristic curve for predicting residual disease

The ROC curve (Fig. 1) was used to predict the residual disease following LEEP for CIN 3. The area under a ROC curve (area under the curve, AUC) of 1.0 corresponded to a 100% accurate outcome prediction. In this study, the point on the curve closest to the upper left corner corresponded to a threshold total risk score of 64.5. The AUC was 0.760 (*p* < 0.001). At this cut-off value, the absolute risk score could predict residual disease with a sensitivity of 67.2% and a specificity of 72.8%.

## Discussion

An estimated 70% of women with a high-grade squamous intraepithelial lesion (HSIL) carry persistent disease, and 10% of the lesions progress to cervical carcinoma within 10 years without treatment for CIN3 [11]. Based on recommendations by the American Society for Colposcopy and Cervical Pathology (ASCCP), once CIN3 is confirmed histologically, the patient should receive cervical conization according to TZ type [12]. It is advised that patients should be continuously monitored to identify recurrent CIN due to unsuccessful treatment, incomplete treatment, or persistent HPV infection after conization [13].

Although the cone margin status of LEEP specimens has been proposed as an accurate predictive factor for residual disease, a free margin does not always indicate complete excision. This is due to the possibility of multifocal cervical lesions, conization with more tissue fragmentation or un-interpretable surgical margins [14]. In this study, 103 (35.40%) patients with a negative margin found a residual lesion in the hysterectomy sample following LEEP for CIN3.

Moore BC et al. reported that residual disease following LEEP could be found in up to 23–31% of patients with negative cone margins and could be absent in up to 37–60% of patients with positive cone margins [15]. Therefore, the margin status in the LEEP sample is not the only predictive factor of post-hysterectomy residual disease. Patients of advanced age and menopausal women who undergo a hysterectomy after initial conization for HSIL are at a significantly higher risk of residual disease than younger patients [16]. For many patients, a hysterectomy following LEEP for CIN3 based on cone margin status alone may not be the optimal treatment. In this study, 55 patients (39.86%) with a positive margin following LEEP for CIN3 had no residual disease in the hysterectomy specimen. The surgery was considered unnecessary.

Multiple factors are thought to increase the risk of significant residual disease after excisional treatment. The odds ratio (OR) for covariates was adjusted in this multivariate logistic regression model. According to this analysis, the factors significantly predictive of residual disease following LEEP included positive ECC, positive margin rate of the specimens from LEEP, type II or III transformation zone, and positive HPV16, HPV18, and other high-risk HPV. Positive HPV-18 tests (OR, 7.13; 95% CI, 3.49 to 14.56; *p* < 0.001) and type III transformation zone (OR, 6.37; 95% CI, 2.91 to 13.94; *p* < 0.001) were the most significant risk factors of residual disease following LEEP.

Kang WD et al. reported that the residual CIN 3 rate in women infected with HPV 16 and HPV 18 at the time of LEEP was significantly higher than in women with other HPV genotypes [9]. All HPV 16, HPV18, and other high-risk HPV resulted in an increased risk of residual disease in the hysterectomy specimen. Furthermore, positive HPV18 status showed the highest risk as being the most potent independent predictive factor of residual disease in a subsequent hysterectomy following a LEEP for CIN 3.

Furthermore, the current study is the first to find that type II or III transformation zone are predictive of residual disease in a subsequent hysterectomy following a LEEP for CIN 3. The cervical SCJ represents where the endocervical columnar epithelium changes to the ectocervical squamous epithelium. The TZ refers to the area between the original and the new SCJ in the cervix [17]. This area is of particular interest due to the high degree of active immature cell proliferation, leading to an increased risk of preneoplastic and neoplastic changes. Type I TZ is completely ectocervical and does not have any endocervical portion. Type II TZ shows an endocervical portion, but the SCJ is completely visible by uncover the cervical canal. In Type III TZ, the border between squamous and columnar epithelium is completely invisible. Therefore, type II or III TZ indicate that the cervical transformation zone is partly visible or not visible. The occult lesion may be located deep in the cervical canal and be hard to detect, which may increase the risk of residual disease in the hysterectomy specimen. About 9.4% patients with CIN3 had skip lesions in the study, which is associated with elevated risk for residual lesion. The term “skip lesion” refers to lesion lying deep in cervical canal discontiguous with other lesions in TZ. Hence, a negative margin on a cone or LEEP can not completely rule out the diagnosis of residual lesions. It is also important to predict and recognize coexistence of residual glandular lesions such as endocervical adenocarcinoma (EAC) and adenocarcinoma in situ (AIS) in hysterectomy specimens, which can prevent delayed treatment [18]. Therefore, cervical cone biopsies performed for squamous cell abnormalities should be thoroughly evaluated for glandular lesions. The clinical prediction model of the current study is based on multiple factors and can predict residual disease in a subsequent hysterectomy. The present clinical prediction model can predict the residual disease in a subsequent hysterectomy following a LEEP for CIN 3 with an AUC of 0.760 (*p* < 0.001). At the cut-off level of 64.5, the total risk score could predict residual disease with a sensitivity of 67.2% and a specificity of 72.8%. Hysterectomy should be considered only in selected cases after carefully reviewing the above risk factors.

This study is limited by its retrospective design and the inclusion of only two medical centers. Furthermore, the duration of HPV infection prior to hysterectomy may be associated with the severity of the disease, but the previous HPV test results were unavailable for most patients. Due to the retrospective nature of this study, some information could not be collected. The data about immunohistochemical pathology and endocervical cytology after LEEP was also incomplete, which may be a risk factor in predicting residual disease in subsequent hysterectomy specimens.

## Conclusions

In summary, the multivariate logistic regression model showed that positive high-risk HPV, particularly HPV18, positive ECC, positive margin of specimens from LEEP, and type II or III transformation zone, were reliable prognostic markers of residual disease following a LEEP for CIN 3.

## Data Availability

The study data are not publicly available due to ethical and legal restrictions. However, data may be made available from the corresponding author upon reasonable request.
